# Investigation of Scaling and Materials’ Performance in Simulated Geothermal Brine

**DOI:** 10.3390/ma17215250

**Published:** 2024-10-28

**Authors:** David Martelo, Briony Holmes, Namrata Kale, Samuel Warren Scott, Shiladitya Paul

**Affiliations:** 1 TWI Ltd., Cambridge CB21 6AL, UK; briony.holmes@twi.co.uk (B.H.);; 2 Institute of Earth Sciences, University of Iceland, 102 Reykjavik, Iceland; 3 Materials Innovation Centre, School of Engineering, University of Leicester, Leicester LE1 7RH, UK

**Keywords:** geothermal energy, calcite scaling, anti-scaling materials

## Abstract

Geothermal energy generation faces challenges in efficiency, partly due to restrictions on reinjection temperatures caused by scaling issues. Therefore, developing strategies to prevent scaling is critical. This study aims to simulate the scaling tendencies and corrosion effects of geothermal fluids on various construction materials used in scaling reactor/retention tank systems. A range of materials, including carbon steel, austenitic stainless steel, duplex stainless steel, two proprietary two-part epoxy coatings, and thermally sprayed aluminium (TSA), were tested in a simulated geothermal brine. Experiments were conducted in a laboratory vessel designed to replicate the wall shear stress conditions expected in a scaling reactor. The tests revealed varying scaling tendencies among the materials, with minimal corrosion observed. The dominant scale formed was calcium carbonate, consistent with geochemical modelling. The findings suggest that despite the high operating temperatures, the risk of corrosion remains low due to the brine’s low chloride content, while the wettability of materials after immersion may serve as a useful indicator for selecting those that promote scaling.

## 1. Introduction

Geothermal energy resources offer a sustainable and eco-friendly form of power generation. However, the efficiency and lifespan of geothermal power plants are often compromised by the phenomena of corrosion and scaling, which can lead to significant operational challenges. Corrosion can take place due to wrong material selection processes or inadvertent changes in the chemistry of the geothermal brine. Scaling occurs when dissolved minerals precipitate from the geothermal fluid and deposit on the surfaces of pipes and equipment, causing blockages and increasing maintenance costs. 

Calcium carbonate (CaCO_3_) is one of the most common scaling minerals encountered in geothermal operations. The scaling mechanism is typically related to boiling, which leads to the transfer of CO_2_ from the liquid to the vapour phase. This reduces the equilibrium partial pressure of CO_2_ in the liquid, increases the pH, and results in a significant increase in the concentration of carbonate ions (CO_3_^2−^) in the aqueous fluid [1,2]. However, the formation of the scales is influenced by various factors, including temperature, pressure, flow rate, and the chemical composition of the geothermal fluid (including inhibitor additives). For example, Muryanto et al. found in their experiments that scaling increased with an increase in flow rate in the laminar regime due to insufficient shear forces to generate detachment [3]. Additionally, other research in this topic [4,5] demonstrated that the same factors mentioned above (which will also play a role in the corrosion of the materials exposed to the brine) have an impact on the type of scaling (stoichiometry, crystalline structure, morphology, among others), highlighting the complex interplay of operational parameters in scaling processes.

The materials used in geothermal systems, such as stainless steel, carbon steel, and duplex stainless steel, play an intermixed role in the corrosion–scaling behaviour in components exposed to the geothermal brine [6], i.e., scaling can reduce corrosion rate. Temperature and some species in the geothermal brine, such as CO_2_, chlorides, H_2_S, ammonia, and hydrogen ions (pH), are found to be key players in the corrosion processes [7,8]. The selection of appropriate materials can mitigate the adverse effects of corrosion, with some coatings being found to be useful to mitigate scaling as well [9]. However, no simple rule of thumb exists to define a material selection for a specific geothermal brine, i.e., carbon steel alloys tend to be used in mild environments when thick wall components are allowed; however, the corrosion rate can increase significantly when pH is below 6, on oxygen ingress into the system, or when chloride concentration is above 2% [7]. Stainless steels are more prone to localised corrosion attack modes, such as stress corrosion cracking, pitting, or crevice, than to uniform corrosion when exposed to geothermal brines, with duplex grades being, in general, more suitable for more demanding environments. In stainless grades, temperature and chlorides are relevant parameters to consider for material selection. Extensive work on the selection of materials for geothermal application has been published by DeBerry and Ellis [7,10,11]. Non-metallic materials are also of use in some geothermal applications, i.e., concrete polymer composites or fibre-reinforced laminates, in some cases due to the natural advantage of some polymers over metals in relation to corrosion resistance; however, some non-metallic materials have been used to reduce scaling, as shown in the next paragraph. 

To address scaling, various methods are currently used in geothermal systems. For example, operational strategies such as controlling fluid temperature can influence scaling tendencies [12]. Experiments have shown that maintaining optimal operational parameters can reduce the rate of scale deposition. Chemical treatments, such as the addition of scale inhibitors, have been widely employed. For example, specific inhibitors like phosphorous-free polyaspartic acids have been shown to be effective in preventing CaCO_3_ scale formation [13]. Research into novel materials and coatings for geothermal components continues to advance, with promising developments in coating materials that resist scaling more effectively, such as polytetrafluoroethylene (PTFE)-blended (polyphenylenesulfide) PPS coating [14].

Although the strategies mentioned previously are implemented currently in geothermal sites and have proved effective, they present some drawbacks. For example, the efficiency of a geothermal site depends on having the minimum possible reinjection temperature; therefore, controlling the temperature of the fluid to avoid scaling could impact efficiency. Using scaling inhibitors could generate other issues, such as corrosion or stress corrosion cracking. In this work, a scaling retention system is used to mitigate scaling; in essence, this is a vessel within the geothermal plant specifically built to allow scaling and adapted to allow easy removal of this scale out of the system. In this equipment, materials that can promote scaling are advantageous.

The work presented in this document is part of GeoSmart, a European collaborative project designed to test solutions to improve the flexibility and efficiency of geothermal heat and power systems. Specifically, this research paper is part of a larger study involving the development of a scaling retention system in the Kızıldere 2 geothermal power plant in Türkiye [15,16]. This paper aims to explore the behaviour of materials in a simulated geothermal brine using flow conditions that approximated the predicted flow conditions within the geothermal brine. Due to the nature of the application, both scale-promoting and scale-preventer materials are of interest in this study. In a second part of this research, coupons made of the same materials presented in this paper were introduced into the retention tank installed in Kızıldere 2; the assessment of these will be performed using the same methodology presented here, and the results will be contrasted in a future part of this investigation.

## 2. Materials and Methods

### 2.1. Simulation of Environmental Conditions and Corrosion Tests

#### 2.1.1. Overview of Test Strategy

**Figure 1:**
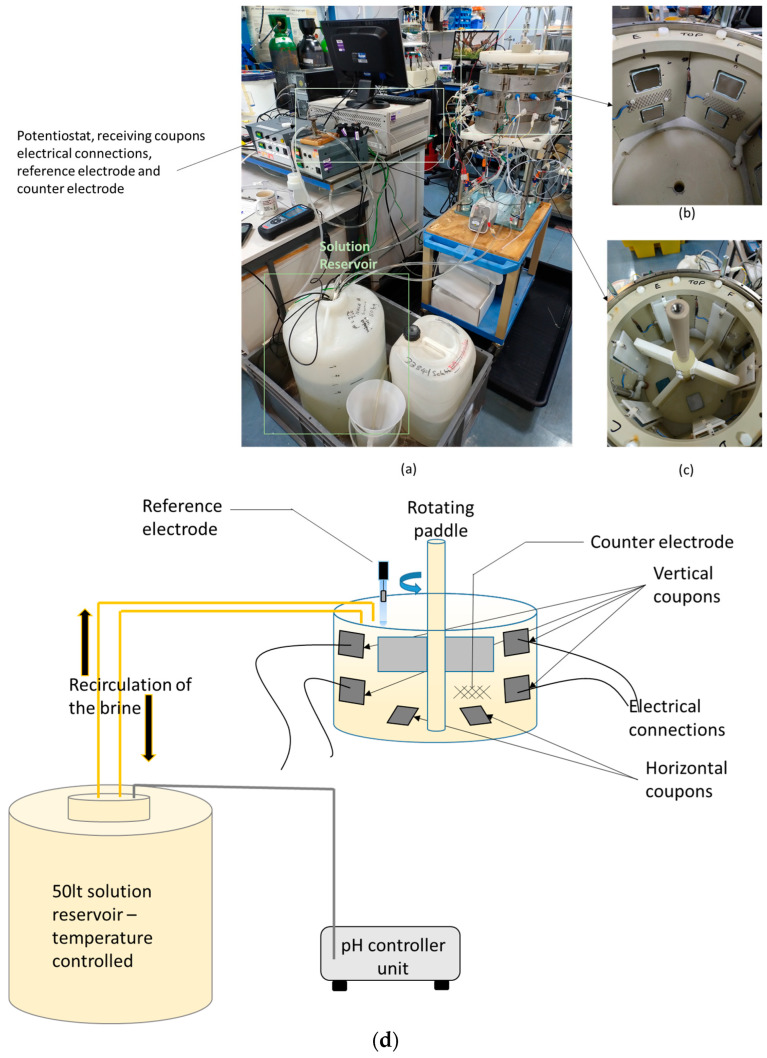
Test rig used for the scaling tests: (**a**) image showing a general view of the rig, with solution reservoirs, small-scale (flow) vessel, and elements required for the electrochemical measurements, such as potentiostats and reference electrodes; (**b**) inside the small-scale vessel, showing the location of the coupons and counter electrodes; (**c**) inside the small-scale vessel, showing the rotating paddle; and (**d**) simplified schematic diagram of main components in the system.

In this investigation, the fluid dynamics and chemical conditions present in the geothermal site of interest were simulated, and coupons of different materials were exposed to these simulated conditions in order to investigate materials’ environmental degradation (corrosion tests). To achieve this objective, a small-scale vessel with rotating paddles was built, the coupons inserted in this vessel were instrumented to measure electrochemical activity (Figure 1 shows images of the built test rig). The targeted component within the geothermal site for the simulation of the flow conditions was the retention tank, which is used for extraction of scaling products from the brine that could cause blockages in the system. The small-scale laboratory testing permitted variables such as material type to be more rapidly assessed than would be possible in the demonstration full-scale retention tank on site in the geothermal plant. 

#### 2.1.2. Simulation of Flow Conditions 

Specifically, the modelling aimed to approximate turbulence levels within the scaling reactor (retention tank) within the fabricated small-scale vessel. Computational fluid dynamics (CFD) analysis was used to understand metrics indicating turbulent mixing in the scaling tank; parameters such as the turbulent intensity, viscosity ratio, and kinetic energy were taken into account. In order to confidently achieve a robust and sensible solution, mesh grid independence studies were undertaken for each geometry. This was primarily carried out by increasing the total number of volume elements in the grid domain and reducing the size of face elements in the regions of interest, particularly near the relevant solid walls. The following assumptions were used for the model:

- Steady state flow: Despite the turbulent mixing process being generally highly unsteady in time and stochastic, a steady flow model provided an initial indicative solution of the forces from the fluid flow acting on the test coupons that can affect (in some way) the scaling process. The steady-state flow assumption was also supported by the low operating speeds in the scaling reactor and small-scale vessel.
- Turbulence modelling approach: The Reynolds–Averaged–Navier–Stokes (RANS) approach was adopted in order to determine the best possible approximation of the velocity and pressure fields around the scaling reactor and small-scale vessel prototypes. This approach implied the use of near-wall functions to model the boundary layer behaviour in the proximity of solid walls without the need to resolve the turbulent scales in the near-wall regions. In this way, the computational effort was significantly reduced together with the complexity of the grid mesh. The shear stress transport (SST) k-w turbulence model in Ansys/Fluent was implemented, owing to the better accuracy in simulating swirling and separated flows compared with the other RANS models (ANSYS, 2022).
- Tetrahedral mesh elements were selected due to their ability to reasonably capture the fluid flow behaviour in complex geometries. For the small-scale vessel, the local mesh size varied from 5.0 mm to 0.5 mm. For the full-size scaling reactor, the face element size in the solid walls of interest was reduced from 22.5 mm to 15 mm across the internal faces and the baffle plates, including the boards and test coupons.
- The temperature was assumed to be constant and uniform.

**Figure 2:**
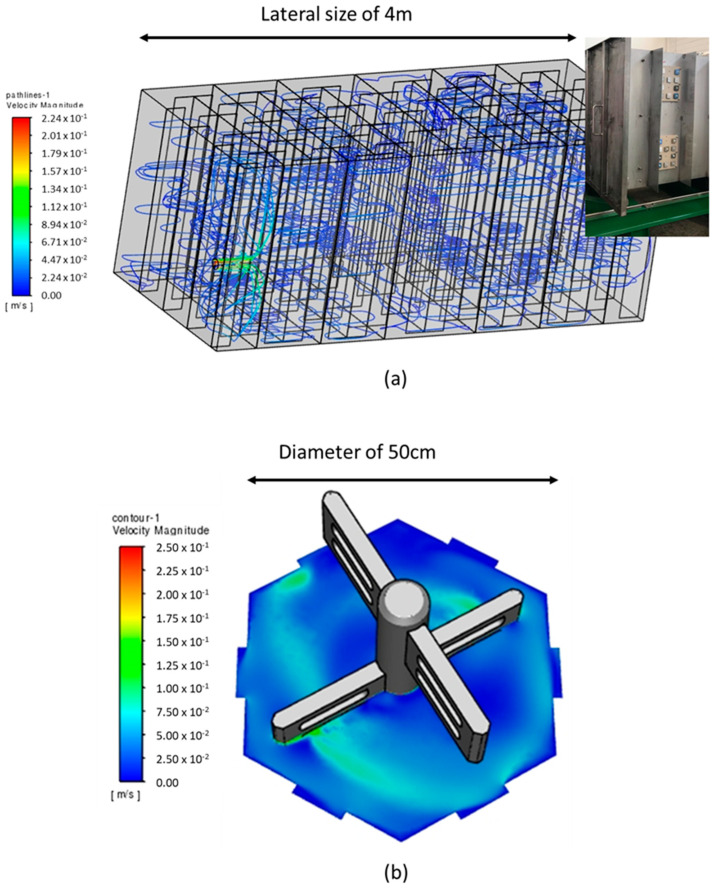
Images generated from the software used for CFD calculations: (**a**) Pathline plot of velocity magnitude through the full three-dimensional domain of the scaling reactor (along with a picture of the coupons installed in the scaling reactor for the field testing) and (**b**) Contour plot of velocity magnitude (m/s) in the small-scale vessel.

Finally, due to the challenges involved in fully replicating the flow complexities from the large retention tank to the small-scale vessel, a one-to-one correlation in flow conditions was not achieved. Specifically, in the small-scale vessel, the main flow-field mechanism is swirling, whereas in the retention tank, separated flow is more common. Wall shear stress (calculated to be at 3.1 mPa in the coupons in the retention tank) was chosen as the key metric for the simulation, as it reflected the tangential mechanical action of the fluid on a solid wall immersed in a flow field. It should be noted that the output of this analysis was the rotation speed of the paddles. Further details of this study can be found in reference [17]. Note: As an indication of the differences in size and geometry of the retention tank and the small-scale vessel, images generated from the software used to perform the CFD are shown in Figure 2.

#### 2.1.3. Brine Chemistry

The chemistry used in the simulated environment was based on the field conditions that were measured at the Kizildere 2 geothermal power plant after the low-pressure separator; see Table 1. The solution composition for the tests was chosen to include the elements that would be corrosive to the metals and alloys (chloride, CO_2_) and/or would be involved in the scaling (Ca, Mg, SiO_2_).

The test temperature was 50 °C, which is the operating temperature in the targeted geothermal system, and should represent a good compromise between scaling and corrosion. Note: This is the temperature that will generate the maximum thermal efficiency in the system, as it can assist in increasing the temperature range of heat energy extraction. Conversely, at this temperature, the scaling tendency should be higher than at a higher temperature, which therefore supports the objective of the scaling retention tank.

For the preparation of the solution, 2 g of NaOH were added to 500 mL of deionised water (DI) water to make a solution of 0.1 M NaOH. Following this, 1.804 g of SiO_2_ were added to the 500 mL solution of NaOH (this was called solution 1). In another flask, 24.2 g of NaHCO_3_ and 7.64 g of NaCO_3_ were diluted in 2 l of DI water (this was called solution 2). When the SiO_2_ was completely dissolved in solution 1, this was mixed with solution 2. Subsequently, 0.5388 g of calcium chloride dihydrate (CaCl_2_·2H_2_O) and 0.7398 g of magnesium chloride hexahydrate (MgCl_2_·6H_2_O) were added to the mixed solution (solution 1 + 2). Last, the final solution was diluted in DI water to give a total of 4 L, and drops of 5 M NaOH were added to adjust the pH to 9.7 at room temperature.

A 50 l solution reservoir was used for the tests, and the solution in this reservoir was transported to the test vessel using a peristaltic pump.

#### 2.1.4. Materials

For this investigation, metallic alloys and different types of coatings were exposed to the brine to determine the effects of the environment. The materials used were: carbon steel S355JR, 304L austenitic stainless steel, lean duplex stainless steel, and the following coatings, defined as: epoxy type coating A (scale preventer), epoxy type B coating (scale promoter), and thermally sprayed aluminium (TSA). The substrate for the coated samples was carbon steel S355JR. The size of the coupons was 40 mm × 40 mm with a thickness of 5 mm. They were fixed inside the small-scale vessel in two orientations: vertical (i.e., on the vessel wall) and horizontal (i.e., on the vessel base). Only the vertical coupons were instrumented for electrochemical measurements. In total, 36 vertical coupons were used during testing (6 of each material) and 17 horizontal coupons. 

#### 2.1.5. Electrochemical Measurements

The monitoring of corrosion activity in the test vessel was carried out using open circuit potential (OCP) and linear polarisation resistance (LPR) measurements. The measurements were conducted using a standardised 3-electrode cell set-up. For the reference electrode, an Ag/AgCl reference electrode was used via a salt bridge to the small-scale vessel, and for the counter electrode, multiple platinised titanium meshes of approximately 40 mm × 70 mm were used. The temperature was regulated in the solution reservoir using a Grant Instruments™ (Cambridge, UK) TX-150 heater. Temperature measurements were carried out in the solution reservoir and in the small-scale vessel. An ACM potentiostat was employed for the monitoring and controlling of current and voltage to carry out the electrochemical testing.

The LPR measurements were conducted every hour, and the polarisation resistance (Rp) was obtained by polarising the working specimens by ±20 mV vs. the OCP. Potentiodynamic polarisation scans were carried out in order to determine the values of the Tafel slopes for the determination of the corrosion rate based on the LPR measurements. Further details about the procedure for determination of the corrosion rate based on the polarisation resistance can be found in ASTM G59-97 [18].

#### 2.1.6. Post-Test Examination of the Coupons

After completion of the test, after approximately 12 days of immersion, the samples were inspected visually to determine scaling and/or corrosion events. Following this, the samples were examined in the scanning electron microscope (SEM) in order to obtain high magnification imaging of the samples, and energy-dispersive X-ray spectroscopy (EDX) was used to perform elemental analyses; different locations within the specimens were examined to confirm consistency among the results. Following this, the samples were examined by X-ray diffraction (XRD).

## 3. Results

### 3.1. Electrochemical Measurements on the Vertical Coupons

**Figure 3:**
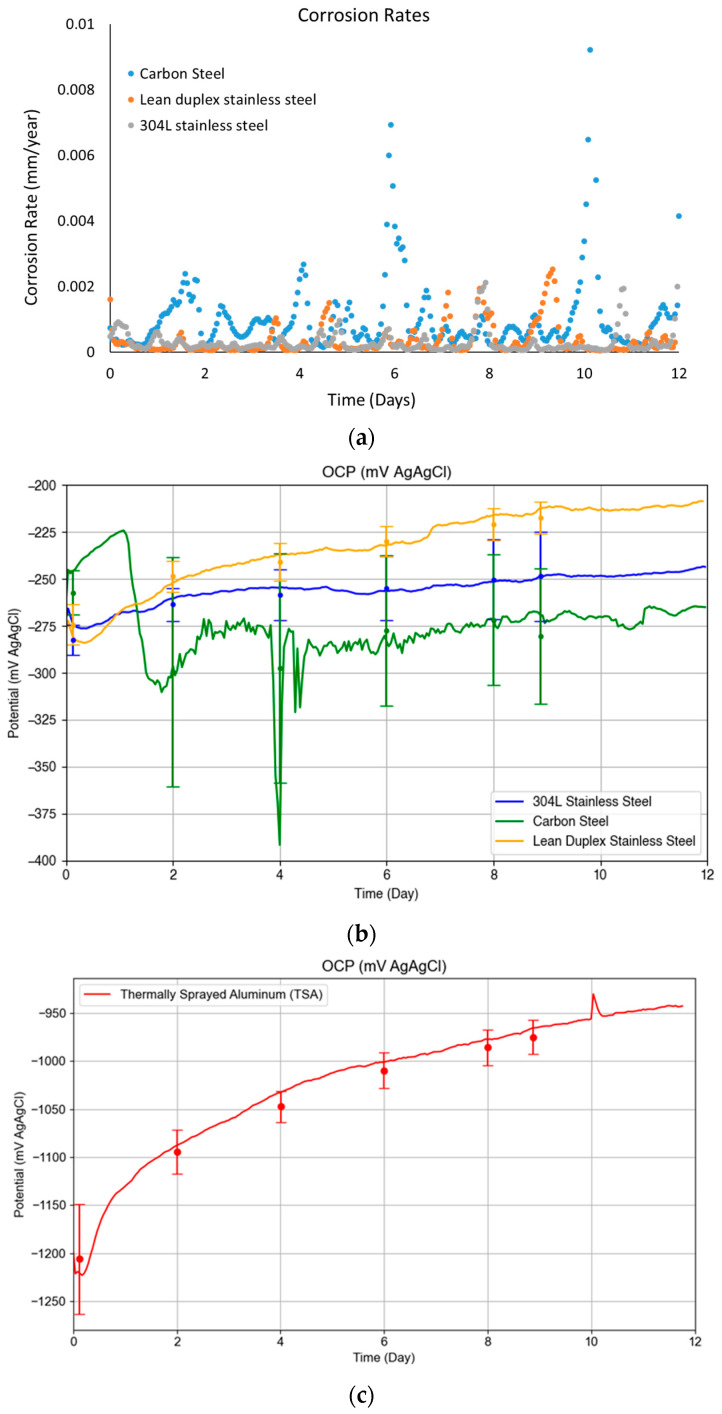
Evolution of the electrochemical measurements across the 12 days of testing in the vertical coupons for the carbon steel, austenitic stainless steel, lean duplex stainless steel, and TSA. (**a**) Corrosion rates obtained from the linear polarisation scans. (**b**) Open circuit potential measurements versus the Ag/AgCl reference electrode, and (**c**) Open circuit potential measurements versus the Ag/AgCl reference electrode for the TSA sample.

Figure 3a shows representative examples of the corrosion rates calculated for coupons of carbon steel and stainless steel from the LPR measurements. As observed from the curves, the corrosion rate tends to be higher in the carbon steel, as expected—this tended to be in the range below 0.002 mm/year, with no peak above 0.015 mm/year. The first peak (between days 5 and 6) was attributed to a very small addition of diluted HCl, which was used to keep the pH at 9.7, as an increase in pH was noted by day 5 of testing; the reason for the second peak is unknown. For carbon steel, depending on the application, different corrosion rate acceptability criteria (corrosion allowance) have been established (user defined) [19]; however, the numbers obtained from tests are indicative, in general, of low corrosion rate. Regarding the OCP measurements, Figure 3b, it can be seen that the initial OCP value was similar for all the materials at around −260 mV vs. Ag/AgCl electrode at the beginning of the tests, and in the case of the carbon steel coupons, this did not show a particular trend throughout the duration of the tests. In the case of the austenitic stainless steel and the lean duplex stainless steel samples, this shifted to a slightly more positive potential by the end of the 12-day test—this variation reflects a change in the cathodic and/or anodic reactions occurring on the surface of the coupons, i.e., formation of scale on the surface of the samples. In the case of the TSA coupons, Figure 3c, the OCP measurements showed an initial drop in potential (more negative) in the first few hours, followed by an increase during the subsequent 12 days. In the case of TSA, it should be noted that due to the porous structure of the coating, if electrolyte penetrates through the coating, the OCP could be the mixed potential of the aluminium coating and the carbon steel substrate. In particular, investigation of TSA in the literature has revealed that the initial drop in potential could be attributed to dissolution of the air-formed film, while the subsequent increase could be attributed to the formation of protective corrosion products or calcareous deposits [20].

In the case of the epoxy coated specimens, the slope of the LPR measurements was consistent with very low corrosion rates, and another feature of these measurements was that the OCP data acquired were noisy—these two features are indicative of no electrochemical activity on the substrate material.

### 3.2. Visual Examination of the Coupons After Testing 

**Figure 4:**
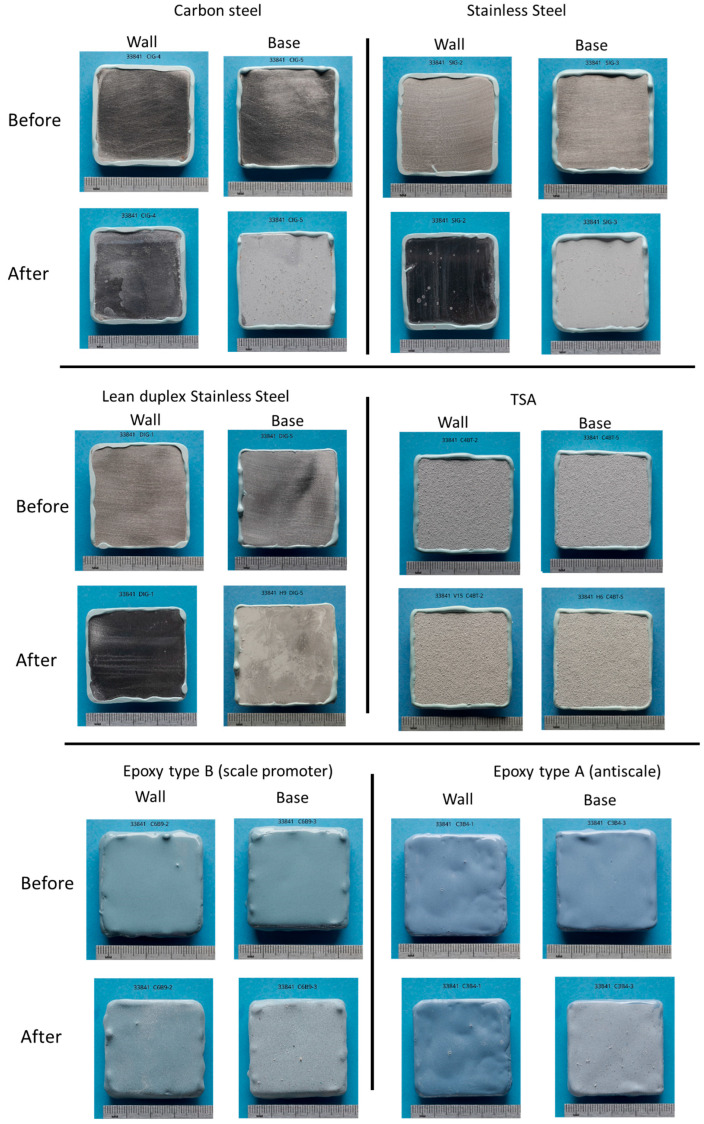
Visual inspection of samples subjected to the exposure tests after 12 days. Starting from carbon steel and stainless steel, followed by lean duplex stainless steel and TSA, and finally the coupons coated with epoxy type B and epoxy type A.

After completion of the 12 days of testing, the samples were removed from the small-scale vessel, rinsed with DI water, and allowed to dry in air. Visual comparison of the vertical coupons before and after testing revealed no significant differences in the appearance of the samples, apart from a minor level of scaling on the surface and that the samples appeared to be more opaque in comparison with the initial shiny appearance of these surfaces (see Figure 4). This opaqueness could also suggest that the changes in OCP could not only be attributed to scaling but also to the influence of the brine on the passive/air-formed film on the materials. For the epoxy-coated samples, the level of scaling on epoxy type B appeared higher than on epoxy type A. In contrast, the entire surface of the horizontal coupons was covered with scales of white appearance. Figure 3 provides images of all analysed coupons. As noted from the images, due to the colour of the deposited scales and the colour and texture of the TSA sample, it was not possible to fully confirm the absence of scales in this sample; however, it would appear that no scaling occurred on this sample.

Furthermore, either based on the visual appearance of the surface of the specimens, i.e., the absence of events of localised corrosion or coating peeling, or based on the colour of the deposited scales, it was considered that the materials used in the tests were not prone to corrosion during the exposure period.

### 3.3. SEM Examination of the Coupons

**Figure 5:**
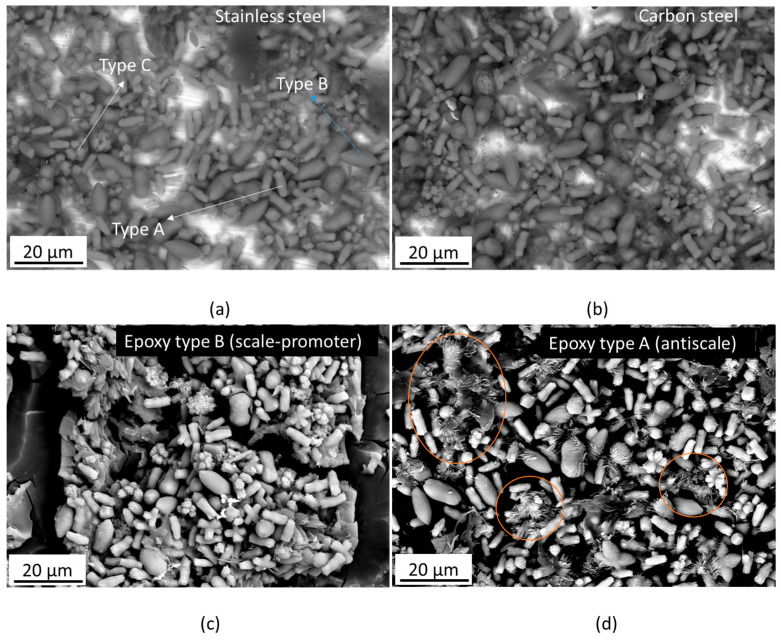
SEM images of the scales taken at a magnification that allowed identification of the individual morphologies of the components of the scale: (**a**) scales on the 304L stainless steel sample, (**b**) scales on the steel sample, (**c**) scales on the epoxy type B, and (**d**) scales on the epoxy type A.

**Figure 6:**
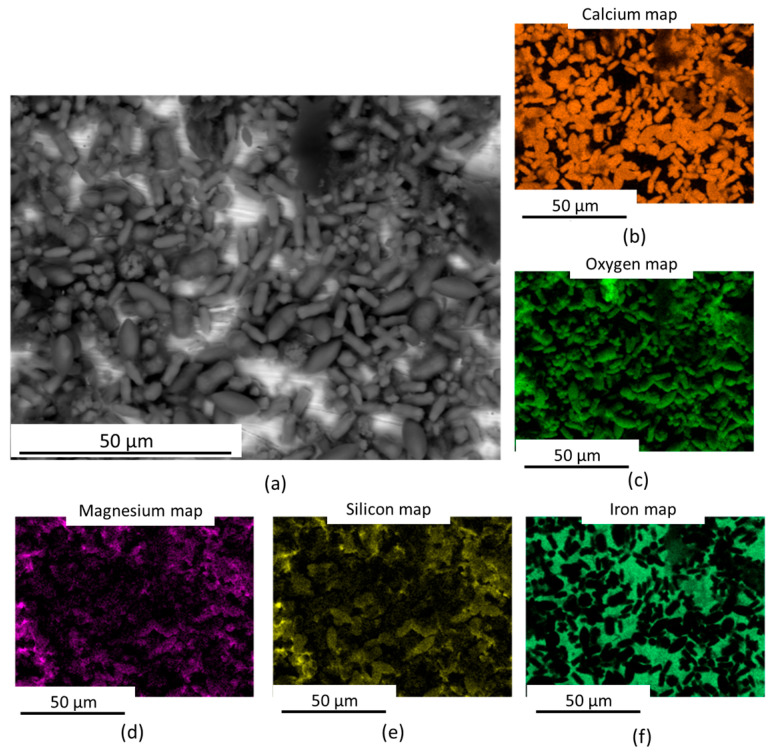
SEM-EDX information obtained from different scales. SEM image and EDX maps corresponding to the scale on the 304L stainless steel sample: (**a**) backscattered electron SEM image of the scale, along with EDX maps of: (**b**) calcium, (**c**) oxygen, (**d**) magnesium, (**e**) silicon and (**f**) iron.

Figure 5 shows SEM images of the scales on two different coupons: carbon steel (Figure 5a) and 304L stainless steel (Figure 5b). The scales formed on both surfaces looked very similar and were composed of features with very specific morphologies, which could be described as a cylindrical curvy shape (type A in Figure 5a), a prolate spheroid “rugby ball” shape (type B in Figure 5a), and a multi-prong shape (type C in Figure 5a). The chemical identification of these from EDX mapping showed that the main elements in the scales were calcium and oxygen (it should be noted that carbon was not included in the maps due to the influence of contaminant elements enriched with this element that could appear in the map). Regarding the presence of silicon in the different types of scales on the surface, it would appear that this element can draw better the silhouette of the type B shape, suggesting Si is associated with type B scale; this is the same case for magnesium. EDX maps exemplifying the behaviour described above can be seen in Figure 6. 

**Figure 7:**
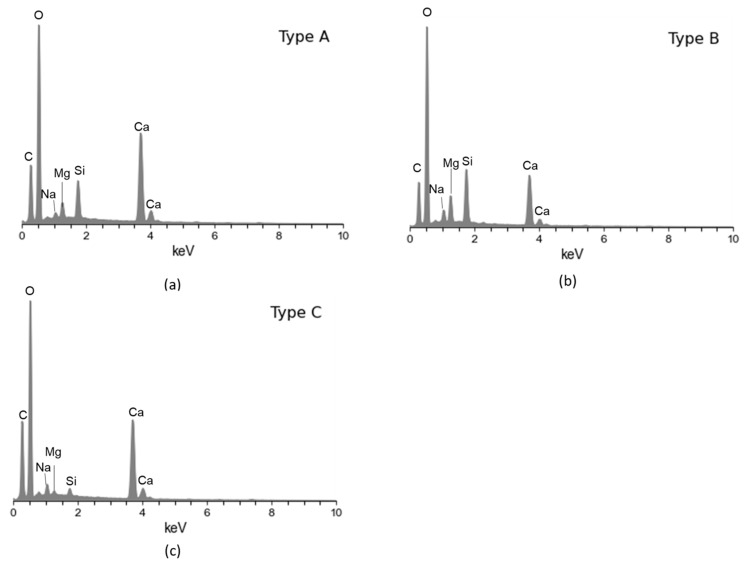
EDX point spectra from scales on the horizontal 304L stainless steel coupon: (**a**) from type A scale from Figure 5a, (**b**) from type B scale in Figure 5a, and (**c**) from type C scale from Figure 5a.

EDX point spectra were taken from each of the scale types, and the spectra confirmed these observations; see Figure 7. Given the absence of iron in the scales or other products from the metallic substrate, we could infer that the scale formation is only associated with the chemistry of the brine and not related to electrochemical reactions in the system, e.g., corrosion. Based on the chemistry of the scales, we could infer that the deposit observed on the surface is a carbonate scale.

Regarding the scales on the coated samples, the precipitates were similar to those observed on the metallic coupons; see examples in Figure 5c for epoxy type B. For the epoxy type A, it appeared that although the morphologies described previously for the metallic coupons were present, some new hairier features can be seen; see highlighted regions in Figure 5d. Regarding the epoxy itself, it appears that in both cases, the exposure to the solution caused cracking on the surface of the coatings. This is attributed to water absorption at the surface of the coatings and is due to differences in stresses with respect to the rest of the coating and substrate. This effect was significantly more pronounced in the epoxy type B (scale promoter coating).

### 3.4. XRD Characterization of the Surface of the Coupons

**Figure 8:**
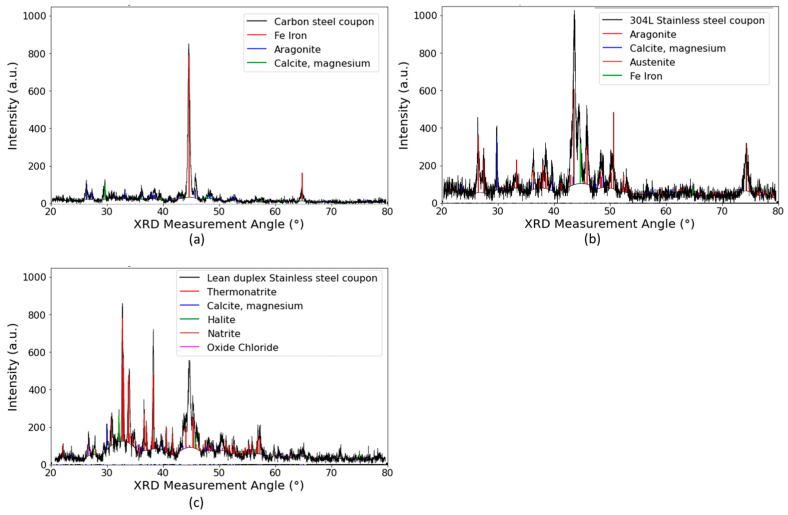
Glancing angle XRD spectra from the surface of the horizontal samples, where the XRD measurement angle corresponded to 2theta. The black spectrum was the obtained experimental data, with library database peaks shown in colour. (**a**) from carbon steel, (**b**) from 304L stainless steel, and (**c**) lean duplex stainless steel.

Spectra of the XRD data collected from the horizontal uncoated samples are shown in Figure 8. For the carbon steel, this displays the expected peaks of ferrite, along with peaks that matched aragonite and calcite; these are typical calcium carbonate scales. It is observed that in the case of calcite, this is matching a magnesium-containing calcite. Based on this information, it could be speculated that calcite corresponded to the type B scale, while aragonite corresponded to the type C scale. In the case of the spectrum obtained from the lean duplex stainless steel coupons, the phase thermonatrite was also found (see Figure 8c).

**Figure 9:**
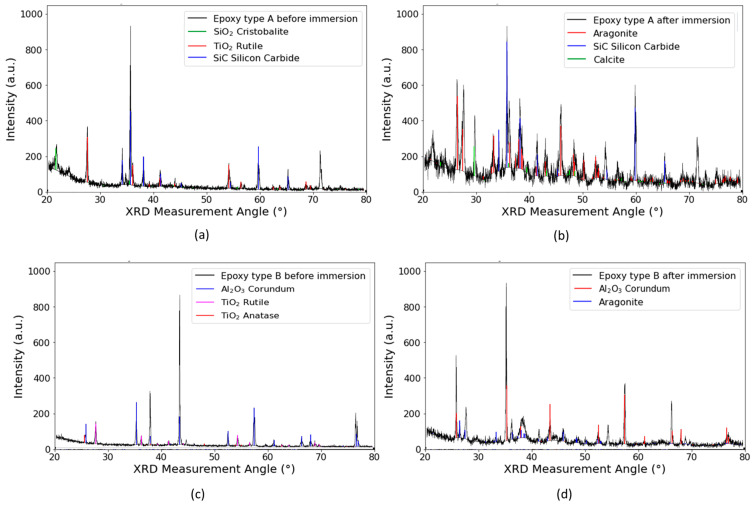
Glancing angle XRD spectra from the surface of epoxy-coated samples, where the XRD measurement angle corresponded to 2theta. The black spectrum was the obtained experimental data, with library database peaks shown in colour. (**a**) from virgin epoxy type A, (**b**) from horizontally immersed coupon of Epoxy type A, (**c**) from virgin epoxy type B and (**d**) from horizontally immersed coupon of Epoxy type B.

On the coated samples, in order to assist with the interpretation of the data, XRD measurements were also performed on un-scaled samples, with the objective being to determine which peaks belonged to the coating substrate. In epoxy type A, the peaks appearing on the scaled sample that did not appear on the substrate sample are highlighted with a star in Figure 9. It was found that all these peaks could be correlated with either aragonite or calcite. In the case of epoxy type B, some peaks appearing in the XRD spectrum of the unscaled sample did not appear in the scaled sample; the reason for this is unknown; however, importantly, the peaks appearing in the scaled epoxy spectrum that did not appear in the substrate spectrum correlated with either calcite or aragonite, indicating that the substrate did not influence the type of scale on the specimens.

**Figure 10:**
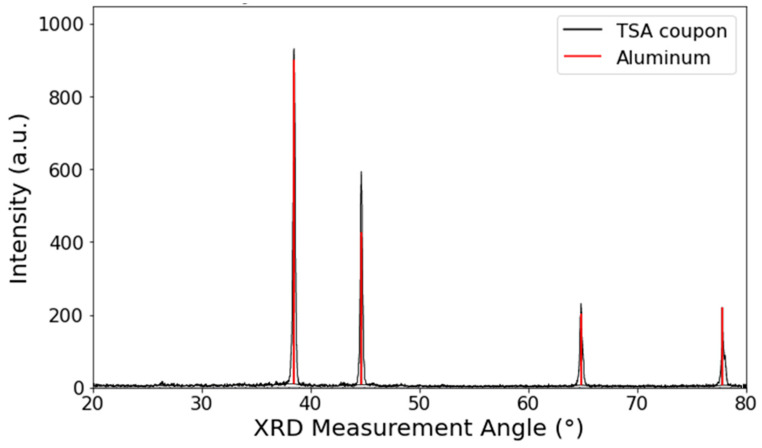
Glancing angle XRD spectra from the surface of the horizontal TSA-coated sample, where the XRD measurement angle corresponded to 2theta. The black spectrum was the obtained experimental data, with library database peaks shown in colour.

The TSA XRD spectrum only showed peaks corresponding to aluminium, suggesting no scaling had occurred on the surface of this sample (see Figure 10).

## 4. Discussion

### 4.1. Solution Chemistry and Precipitation

The chemistry of the geothermal brine was modelled using the GEM-Selektor v3.1 software package [21,22] employing the PSI-Nagra 12/07 thermodynamic database [23]. The modelling calculations used the chemical composition of the brine listed in Table 1 and aimed to predict the saturation state of various scaling minerals under the simulated geothermal conditions.

**Figure 11:**
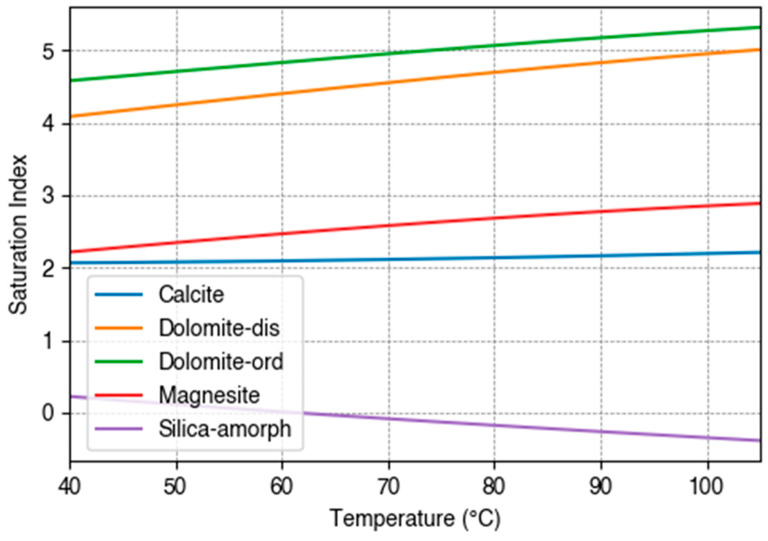
Saturation indices of selected minerals as a function of temperature for the simulated geothermal brine. The saturation index (SI) is defined as SI = log_10_(Q/K), where Q is the ion activity product of the mineral in solution and K is the equilibrium constant for the mineral’s dissolution reaction. A positive SI value indicates that the mineral is supersaturated and likely to precipitate, while a negative SI value suggests undersaturation.

As illustrated in Figure 11, the modelling results indicate that several carbonate minerals, such as calcite, dolomite, and magnesite, are expected to be supersaturated under the experimental conditions, as reflected by their positive saturation indices. This modelling result aligns well with the experimental results indicating calcium carbonate scale formation. It is noteworthy that the saturation index for amorphous silica is only slightly positive at the experimental temperature of 40 °C, indicating a low propensity for silica precipitation. While this is somewhat unexpected, given the typically high concentrations of silica in geothermal brines and its common association with scaling issues, the lack of significant silica precipitation observed in our study could be attributed to the elevated pH of the geothermal brine, which leads to significant hydrolysis of silicic acid and increases the solubility of silica in solution [15]. While the saturation index for amorphous silica becomes negative with increasing temperature, the observed trend of increasing saturation index with increasing temperature for the carbonate minerals supports the likelihood of carbonate scaling under higher temperature conditions.

The work of Thomas Lovering suggested that the formation of dolomite is a function of the ratio of calcium to magnesium and temperature [24]. The results presented in Section 3.3 and Section 3.4 suggest that the calcite precipitated on the coupons was rich in magnesium, therefore altering the ratio of calcium and magnesium in the system, and this is perhaps the reason for the slight difference between the geochemical modelling and laboratory test results.

### 4.2. Materials Corrosion Behaviour

The tests conducted in this program, and described above, have revealed that the brine used for testing does not produce significant corrosion of the materials tested: resulting in either a low corrosion rate in the case of the carbon steel or no corrosion events in the case of the stainless steels, with no visible degradation in the coated specimens. Note: in the case of the carbon steel specimens, due to scaling of the specimens, which might cause a reduction of the effective area exposed to the electrolyte, it is not simple to say that the corrosion rate remained constant throughout the test duration, as suggested in Figure 2a, but, in any case, the current measurements and associated corrosion of the specimens were low.

A point to consider is that the tests performed during the investigation were conducted at the retention tank/scaling reactor`s intended operating temperature (50 °C). However, the system may face more severe conditions, especially concerning corrosion. For instance, the inlet temperature to the retention tank could be up to approximately 104 °C, and the pH of the fluid could be altered to increase or decrease the scaling tendency as a function of the brine re-injection conditions. To fully understand the expected level of corrosion of the materials used for this system and the influence of the brine, some further corrosion tests were conducted. Table 2 shows a summary of some of these tests. The results reveal that even at more aggressive conditions, for example, lower pH or higher temperature, the susceptibility to corrosion of the materials tested is not significantly higher. No tests are presented from the other metallic samples, as duplex stainless steels tend to have better corrosion resistance than the conventional austenitic stainless grades, such as 304L. For carbon steel, it is expected that the extent of the corrosion rate increases with temperature in this temperature range.

**Figure 12:**
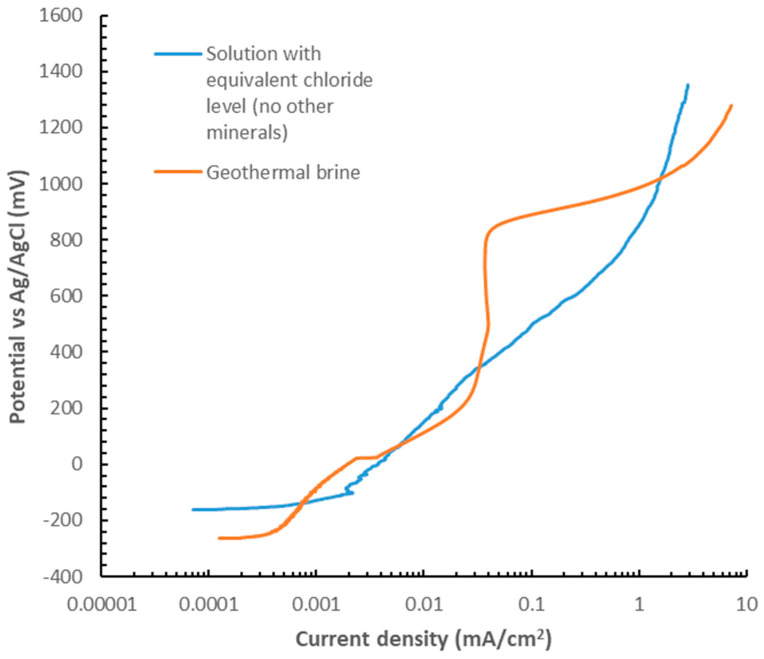
Potentiodynamic polarisation curves of alloy 304L with non-metallic crevice formers in two different solutions at 50 °C and pH 9.7. Solution 1: geothermal brine as described in Table 1. And Solution 2: prepared using only NaCl (apart from use of NaOH to increase pH to 9.7) to obtain the same levels of chlorides as shown in Table 1.

Two possible scenarios could be perceived to explain the reduced corrosion in the materials tested. It could be that the concentrations of ions, particularly chloride, were very low in this solution and no corrosion should be expected, or that the brine solution itself could be acting as a “corrosion inhibitor”. It should be noted that these two hypotheses reflect a very simplified scenario of the complex pitting corrosion mechanism in stainless steel, where the different testing variables could affect the pitting behaviour, i.e., the shape of the pits, the potential for stable pit growth vs. re-passivation, the frequency of pitting, the pit growth rate, among others [25]. The main objective of this exercise was to have a general view of the role of chlorides in promoting corrosion vs. the other elements in the brine. In order to prove this second hypothesis, tests were conducted under the same conditions using the simulated brine, as detailed in Section 2.1.3, and in a solution with an equivalent level of chlorides but without other elements. For these tests, potentiodynamic tests of crevice samples were performed for the 304L stainless steel. The results are shown in Figure 12. The tests were performed on samples with crevice formers. From these curves, it can be seen that apart from a bump in the range from ≈ 120 mV vs. SCE to ≈300 mV vs. SCE, the current is typically higher in the solution containing only chlorides, and in fact, in the geothermal brine, the material displays a semi-passive region. These results suggest that minerals in the brine play an inhibitory role in the localised corrosion of the 304L stainless steel.

### 4.3. Understanding Role of Materials on Scaling

Further examination of the scales was carried out in order to determine the adhesion strength of the scale to the material’s surface. This exercise was carried out using standard coating adhesion protocols, such as tape adhesion or pull-off testing; however, these trials were not fully successful. From these, it was noted that the scales were not strongly adhered to the samples. Taking advantage of this, for some horizontal coupons, the scales were carefully removed (to avoid damaging the substrate) in order to identify scale thickness as a method to compare scaling tendency between the different materials. These measurements were performed on the horizontal coupons, and the results are presented in Table 3. Although some differences were observed, these were not statistically significant, apart from those of 304L stainless steel. The analysis of the horizontal coupons suggests that once a scale starts to form, this will continue, and the role of the material in scaling is less relevant. The smaller thickness of the scale on the 304L stainless steel, which is in agreement with the lower variation in OCP throughout the test compared to the lean duplex stainless steel, might suggest that electrochemical interactions (or local surface chemistry variations in the brine) play a role in the formation of the scale (at least when scaling thickness is still in the short range).

From the visual observations of the vertical coupons, it was noted that the scaling tendency changed across the different coupons. In order to identify a possible reason for this, wettability measurements were performed (water contact angle measurements), and the results are presented in Table 4. Although no one-to-one correlation was found between scaling tendency and water contact angle, for the epoxy coated samples, the epoxy type A (which displayed a higher water contact angle after testing) had less scaling tendency than the epoxy type B. In the stainless steels, the contact angle reduced after exposure to the solution, in agreement with the scaling tendency of the materials, which showed that the scaling tendency of the stainless steels was similar to Epoxy type B, despite the initially higher water contact angle. In the case of TSA, it should be noted that the results suggest that at the beginning of the tests, the water contact angle was very high, and this was attributed to the presence of a high temperature oxide following the thermal spraying process, and then the subsequent, much lower, contact angle was attributed to hydrolysis of this oxide [26]. Therefore, the wettability of the surface could not be used to explain the low scaling tendency in the TSA samples. In addition to this, literature on this topic suggests that TSA tends to be frequently associated with calcareous deposits in subsea applications due to the oxygen reduction (cathodic reaction that causes a local increase in pH) associated with the substrate material [27]. The reason why the measurements performed in this investigation did not show scaling on the TSA specimens is unknown, and it is possible that this is simply a short-term effect: longer testing, as will be carried out in part 2 of this research in the field coupon tests, should be carried out to corroborate this observation. 

According to literature, there are other mechanisms and parameters, apart from wettability (surface tension), that can affect the scaling tendency of materials. In 2023, Fanicchia and Karlsdottir published a comprehensive review of coatings and paints in geothermal systems [28]. In epoxy systems, it was found by Boersma et al. [29] that scaling is governed by Youngs modulus, surface tension (wettability), and roughness. Wang et al. found that the scaling tendency was reduced due to the release of metallic ions from the epoxy coating surface that could combine with the other elements in the brine, causing precipitation of the elements producing scaling in the brine itself rather than on the surface of the coatings. It is not possible to assess these hypotheses in this work. However, it is curious to find that in other systems, scaling increased with increasing roughness, which is contrary to the case of TSA in our investigation [30].

## 5. Conclusions

In this investigation, the impact of a geothermal brine on five different materials was investigated for the GeoSmart project with the intention to understand materials behaviour in a geothermal brine compatible with Kizildere 2 geothermal site. This study focused on the environmental conditions prevalent in the scaling reactor. The analysis of literature on the topic revealed that there are different strategies to mitigate scaling in geothermal systems, including using specific coatings. However, no detailed studies have been performed in laboratory conditions and field tests to assess the scaling of materials, in particular towards pro-scaling materials. This research shows the results of tests conducted under controlled laboratory conditions to replicate the effect of variables considered relevant for the target application: brine chemistry, temperature, and flow.

Under the studied conditions, it was found that the main issue in the materials studied was scaling of carbonate scales (carbonate vs. silica). It was noted that the scaling magnitude could be controlled through the use of different substrate materials. For example, epoxies with targeted chemistry can be used as scale promoter materials. It should be noted that gravitational effects play a significant role in scaling. There was a good correlation between scaling precipitates from laboratory tests and predictive geochemical modelling, which could be used to explain the absence of silica scaling under studied conditions. Regarding corrosion, this work identified that the minerals in the brine could decrease the corrosion current, which could explain why the brine corroded the materials less severely than expected based on chloride content and temperature.

One of the most notorious results of the tests performed was the lack of scaling on the surface of the TSA coupons. This was not expected despite the very high initial hydrophobicity of this coating, which dropped significantly after immersion. One of the reasons for this could be due to the fixed chemistry of the brine and/or the exposure time. In the next stage of this work, the samples inserted in the scaling reactor will be studied using the same analytical tools presented in this work.

## Figures and Tables

**Table 1:** Basis of brine composition for corrosion tests.

Constituent	Brine, mg/L
pH/23 °C	9.7
SiO_2_	451
Na	2773.2
Ca	36.7
Mg	22.1
Cl	129.4
CO_2_	1081.9

**Table 2:** Electrochemical immersion tests. Assessment of crevice and stress corrosion cracking in geothermal brine in 304L stainless steel.

Testing	Material	Brine	Temperature, °C	Results
Exposure tests	304L	Simulated geothermal brine—5bar no oxygen	104	No corrosion events
U-bend tests	304L	Simulated geothermal brine—5bar no oxygen—pH 5 and pH 9.7	104	No SCC -Only few pits were seen in the sample
Crevice tests	304L	Simulated geothermal brine—5bar no oxygen	104	No crevice corrosion, only some pits were found in the specimens

All tests were performed in stagnant solution.

**Table 3:** Measurements of the average scale thickness for the different coupons.

Specimen ID	Step Height			Average	Ra
	M1	M2	M3		
Carbon steel	8.06	8.17	5.42	7.22	1.55
Epoxy type A	4.87	6.13	7.44	6.14	
Epoxy type B	3.63	7.27	14.28	8.39	5.41
304L Stainless	4.45	3.15	3.01	3.54	0.79
Duplex	7.71	12.08	9.15	9.65	2.23
TSA	Could not distinguish between scaled and cleaned regions, surface roughness is quite high				

**Table 4:** Measurements of the average water contact angle for the different coupons.

Specimen ID	Water Contact Angle (^o^)	
	Before	After
Carbon steel	38.5	38.6
Epoxy type A	83.0	72.2
Epoxy type B	79.2	48.3
304L Stainless	104.3	61.9
Duplex	97.5	47.8
TSA	131.4	0

## Data Availability

The original contributions presented in the study are included in the article, further inquiries can be directed to the corresponding authors.
